# Buffering against exposure to mental health misinformation in online communities on Facebook: the interplay of depression literacy and expert moderation

**DOI:** 10.1186/s12889-023-16404-1

**Published:** 2023-08-18

**Authors:** Nicole Bizzotto, Gert-Jan de Bruijn, Peter Johannes Schulz

**Affiliations:** 1 Faculty of Communication, Culture and Society, Università della Svizzera italiana, Lugano, Switzerland; 2 Department of Communication Studies, University of Antwerp, Antwerpen, Belgium; 3 Department of Communication & Media, Ewha Womans University, Seoul, South Korea

**Keywords:** Online communities, Misinformation, Mental health, Depression literacy, Expert moderation

## Abstract

**Background:**

The proliferation of health misinformation on social media is a growing public health concern. Online communities for mental health (OCMHs) are also considered an outlet for exposure to misinformation. This study explored the impact of the self-reported volume of exposure to mental health misinformation in misinformation agreement and the moderating effects of depression literacy and type of OCMHs participation (expert vs. peer-led).

**Methods:**

Participants (n = 403) were recruited in Italian-speaking OCMHs on Facebook. We conducted regression analyses using PROCESS macro (moderated moderation, Model 3). Measures included: the Depression Literacy Questionnaire (Griffiths et al., 2004), the self-reported misinformation exposure in the OCMHs (3 items), and misinformation agreement with the exposure items (3 items). Whether participants were members of expert or peer-led OCMHs was also investigated.

**Results:**

The final model explained the 12% variance in the agreement. There was a positive and significant relationship between misinformation exposure and misinformation agreement (β = 0.3221, p < .001), a significant two-way interaction between misinformation exposure and depression literacy (β = − 0.2179, p = .0014 ), and between self-reported misinformation exposure and type of OCMH (β = − 0.2322, p = .0254), such that at higher levels of depression literacy and in case of participation to expert-led OCMHs, the relationship misinformation exposure-misinformation agreement was weaker. Finally, a three-way interaction was found (β = 0.2497, p = .0144) that showed that depression literacy moderated the positive relationship between misinformation exposure and misinformation agreement such that the more misinformation participants were exposed to, the more they agreed with it unless they had higher levels of depression literacy; this, however, occurred only if they participated in peer-led groups.

**Conclusions:**

Results provide evidence that the more members reported being exposed to mental health misinformation, the more they tended to agree with it, however this was only visible when participants had lower depression literacy and were participating in peer-led OCMHs. Results of this study suggest that both internal factors (i.e., high depression literacy) and external factors (the type of online community individuals were participating in) can buffer the negative effects of misinformation exposure. It also suggests that increasing depression literacy and expert community moderation could curb the negative consequences of misinformation exposure related to mental health. Results will guide interventions to mitigate the effects of misinformation in OCMHs, including encouraging health professionals in their administration and implementing health education programs.

**Supplementary Information:**

The online version contains supplementary material available at 10.1186/s12889-023-16404-1.

## Background

### Health misinformation and the illusory truth effect

Due to the increasing popularity of the internet [1, 2], more specifically of social media [3] as a venue for seeking and sharing health information, there is a growing concern about the spread of health misinformation [4, 5]. Recently, these concerns have intensified due to the COVID-19 pandemic [6]. A recent systematic review of reviews found that the prevalence of health-related misinformation on social media ranged from 0.2 to 28.8% [7].

Swire-Thompson and colleagues [8] define misinformation as “information that is contrary to the epistemic consensus of the scientific community regarding a phenomenon”. Health misinformation is a specific type of misinformation that refers to a “health-related claim of fact that is currently false due to a lack of scientific evidence” [9]. As users generate information on social media, it can be subjective or inaccurate, therefore a worrisome source of health misinformation [6, 10] as it can also be archived and persist over time until it is corrected or deleted, becoming a dangerous resource for future health information seekers [11].

Public health researchers and practitioners are increasingly preoccupied with the potential for health misinformation to misinform and mislead the public as it not only creates erroneous health beliefs confusion and reduces trust in health professionals but can also “delay or prevent effective care, in some cases threatening the lives of individuals” [5, 10]. Thus, combating its effects has become crucial for public health [9, 12] and can be accomplished only by understanding its psychological drivers [13] and complementary buffers. One particularly prominent finding that helps explain why people are susceptible to misinformation is the ‘illusory truth effect’, according to which repeated information is perceived as more truthful than new information [14–18].

In the context of the illusory truth effect, it has been found that the effects of repeated exposure to misinformation on perceptions of accuracy disappeared when the receiver knew the actual truth and that people were more likely to believe misinformation when they were unfamiliar with the issue at hand [19]. Other studies have shown that knowledge is key to buffering against misinformation exposure [20–22].

### Mental health misinformation: underestimated and understudied

According to a recent systematic review, previous studies on health misinformation on social media have concentrated more on topics of physical-related illnesses such as vaccines (32%), drugs or smoking (22%), non-communicable diseases (19%) and pandemics (10%) [23]. Few studies have examined misinformation regarding mental health specifically, although this might be crucial for two reasons. Firstly, mental health is a growing public health concern [24], which has been underestimated even though about 14% of the global disease burden has been attributed to neuropsychiatric disorders such as depression [25]. Secondly, mental health conditions are frequently stigmatized and misunderstood, resulting in a greater prevalence of misinformation online and offline [26–28]. Therefore, it is crucial to understand the conditions that can mitigate the outcomes of mental health misinformation exposure.

### Mental health misinformation in online communities

Online health communities are virtual platforms where individuals (or caregivers) with similar health conditions or concerns gather to share information, seek support, and engage in discussions related to health. These communities are typically specific to a specific illness and are highly prevalent for chronic and marginalized diseases [29], such as mental health disorders. Although these communities can exist in different forms such as forums [30] or as social media groups such as on Facebook [31, 32] or Reddit [33] they all share similar affordances [34] such as the question-and-answer format. Online communities generally rely on the work of volunteers to police themselves [35], some of them being health professionals, others peers with no expert credentials [31, 36, 37]. Although healthcare professionals play a critical role in ensuring information quality in online health communities [38, 39], the literature on this topic needs to be more extensive, especially regarding the differences that these two types of groups might perform, particularly about misinformation.

Online communities for mental health symptoms (OCMHs) are communities specific for mental health topics that serve as virtual spaces where individuals suffering from mental health conditions (or their caregivers) can connect with others experiencing similar challenges. These communities cater to a wide range of mental health topics, including general discussions about mental health [40] as well as more specific topics such as depression and anxiety [33, 41].

OCMHs are becoming increasingly present on social networking sites, especially among younger generations [42] and they can also be considered an outlet for misinformation. A recent content analysis [43] has found extremely high levels of misinformation in OCMHs, and even communities moderated by health professionals (expert-led) were not exempt from this issue. This is in line with other studies showing that healthcare professionals can also spread misinformation in various ways [10].

### Research gaps and current study

The present study aims to investigate the relationship between exposure to mental health misinformation in Italian online communities for mental health on Facebook and related agreement, focusing on two aspects that might impact this relationship and the interplay between them: depression literacy and type of OCMHs moderation.

Health literacy is at the heart of any discussion of health-related misinformation, which can be defined as “the degree to which people have the capacity to obtain, process, and understand basic health information and services needed to make appropriate health decisions” [44]. In a systematic review, results indicated that low health literacy was negatively related to the ability to evaluate online health information [45]. Previous studies have shown that knowledge moderated the relationship between exposure and beliefs but focused on other aspects of literacy, such as news literacy [46] or media literacy [47]. One study found that a higher level of cancer literacy helped participants identify misinformation and prevented them from being persuaded by it [48].

As in the health context, different types of literacy exist in different contexts; in the present study, we focused on declarative knowledge about depression literacy, depression being one of the most common mental illnesses in Italy [49, 50] and worldwide [51]. Depression literacy [52] is a facet of the mental health literacy concept, the latter defined as an individual’s knowledge regarding mental health [53], but with a specific focus on depression intended as a major depressive episode. Mental health literacy, or lack thereof, has been used as a possible factor to explain uncertainties or lack of knowledge about mental health and the ensuing effects on effective treatment and care [54]. Determining whether depression literacy levels can buffer the effects of misinformation exposure is critical, also as identifying which segments of the population are especially vulnerable to health misinformation and developing interventions for individuals at risk.

However, there may be more effective approaches than focusing on individual differences in susceptibility to misinformation while ignoring other potential external factors. As we mentioned earlier, OCMH typologies can vary depending on their moderators’ expertise (peers or mental health experts) and this factor might influence the relationship between exposure and agreement with misinformation. Content moderation scholars posit that content moderation “has exploded as a public, advocacy, and policy concern” [55]. Typically platforms such as Facebook use a combination of algorithmic tools, user reporting and human review [56]. Previous research on misinformation has primarily focused on automated content moderation implemented by platforms through machine learning classifiers [57, 58] and has demonstrated the effectiveness of platforms’ content moderation practices in mitigating the spread of conspiracy theories and other forms of misinformation [59]. However, the effects of human moderation have not been investigated extensively, especially in the context of mental health misinformation, a type of misinformation that poses unique challenges as the complexity of mental health issues requires nuanced approaches to content moderation. However, until now, the literature has predominantly focused on examining the social effects of moderation within health communities (e.g [60, 61]), rather than its significance in countering misinformation. Previous research has in fact demonstrated that the knowledge and guidance provided by peer patients differed significantly from that offered by professional healthcare providers [62].

Therefore, it is critical to investigate potential differences between these groups in misinformation exposure outcomes. Given that the quality and accuracy of online health information provided by OCMHs can vary significantly [63], it is crucial to consider the potential implications of participating in OCMHs with different content moderation types, as this may affect the degree of exposure to health-related misinformation and its associated consequences.

Furthermore, the interplay between internal (depression literacy) and external (type of OCMHs moderation) factors sheds light on the most vulnerable individuals within online communities. This approach will expand upon previous research on individual differences in misinformation susceptibility [16, 22] and provide a more comprehensive understanding of the complex dynamics that may influence agreement with misinformation exposure within OCMHs.

To the best of our knowledge, no study has examined whether and how individual differences such as depression literacy might interact with the external factors embedded in online communities, including the type of moderators’ expertise. However, it is crucial to explore their potential interaction as previous research highlighted the heterogeneous nature of users within online communities, with varying levels of health literacy [64]. Additionally, it is plausible that different types of guidance provided by moderators, depending on their expertise levels, might be less or more able to mitigate the effects of poor literacy, particularly when users are exposed to high volumes of misleading content.

Understanding how depression literacy and moderator expertise interact in online communities can provide valuable insights into optimizing mental health support and interventions. By exploring this interaction, we can identify effective strategies for addressing the challenges posed by varying levels of health literacy and the diverse expertise of moderators. This research can inform the development of targeted interventions and improve outcomes for individuals seeking support for depression within online communities.

We have chosen to focus our research on Italian-speaking OCMHs on Facebook as OCMHs are transitioning from to forums social networking sites [42]. Furthermore, while Facebook’s overall usage worldwide is declining, particularly among younger generations [65], it remains one of the most widely used social networking platforms in Italy as in 2022, 77.5% of surveyed Italian internet users reported using Facebook [66]. Moreover, differently from other Italian-speaking forums or mental health subreddit communities, we have observed a vast amount of active OCMHs communities on the Facebook social media platform, making it a suitable case study. Furthermore, as claimed by Bayer and colleagues [67], as social media will continue to iterate, emerging research can extrapolate the findings from one platform to other platforms such as that “even when a platform is decommissioned, findings linked to its elements can be compared to future channels that share characteristics within the same element”. In other words, we believe that our findings could have the potential to extend beyond the specific OCMHs under study and be used to build research also across other social media platforms.

### Hypotheses and research question

**Fig. 1:**
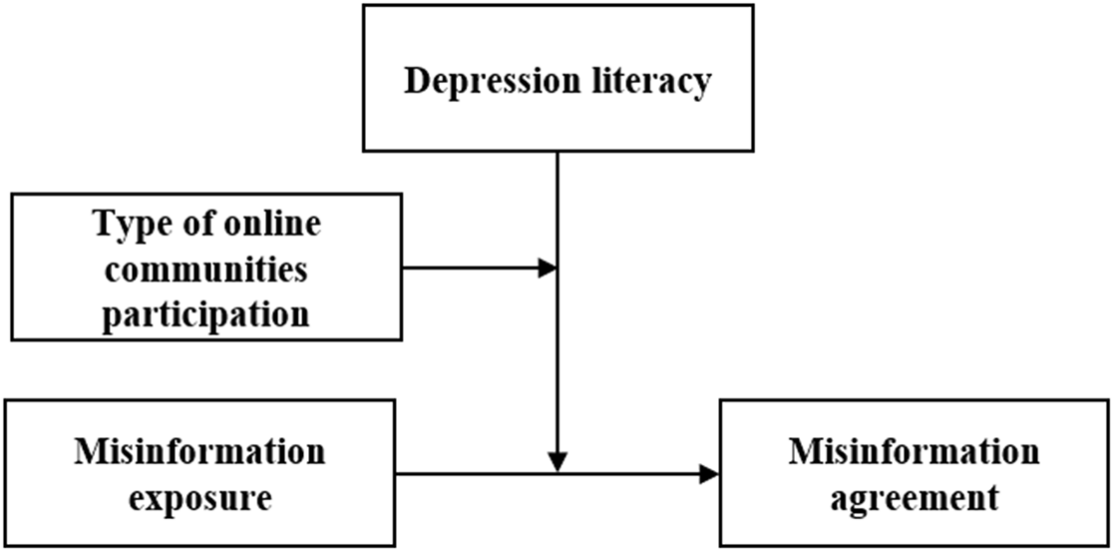
Hypothesized model

We tested the following hypotheses using a moderated moderation model (see Fig. 1 for the hypothesized model). Model 3, as proposed by Hayes [68], is a statistical model used to analyze moderated moderation effects that extends the traditional moderation analysis by examining the interactive effects of two moderating variables on the relationship between an independent variable and a dependent variable. The model allowed us to examine the interaction effects between the two moderating variables (1) depression literacy (individual difference) and (2) type of OCMHs (external factor) on the relationship between the independent (misinformation exposure) and dependent (agreement with misinformation) variables.

First, based on the literature on the Illusory truth effect [69], we expect that:

#### H1

Misinformation exposure will be positively and significantly associated with misinformation agreement.

However, based on the above literature on the influence of the protective role of knowledge [46–48] we hypothesize that:

#### H2

The positive association between misinformation exposure and misinformation agreement will be moderated by depression literacy.

Furthermore, we expect that:

#### H3

The positive association between misinformation exposure and misinformation agreement will be moderated by type of OCMHs participation.

Then, as a research question, we will test whether depression literacy and type of OCMHs participation also interact with each other in the following way:

#### RQ

The moderating effect of depression literacy on the relationship between misinformation exposure and misinformation agreement will be further moderated by the type of OCMHs participation.

## Methods

### Participants and procedure

The data for this study were collected through an online survey on Qualtrics in spring 2022. To recruit participants, the principal investigator reached out to administrators of 72 Italian-speaking OCMHs, all identified by entering mental health-related keywords into the Facebook search bar. OCMHs included in the study could have been moderated by mental health professionals (expert-led) or peer-led. In total, 65% of OCMHs (n = 51) agreed to participate, while 12% (n = 9) declined, and 15% (n = 12) did not respond. Participants were recruited through a video presentation shared on the collaborating OCMHs, including a questionnaire link on Qualtrics. They were rewarded with a financial incentive and access to artistic/poetry videos.

At the beginning of the Qualtrics survey, after providing the informed consent, participants were presented with items related to sociodemographic variables. Subsequently, they were asked to select the specific online mental health group in which they had participated in the previous month from a provided list. Following this, participants were asked to indicate the frequency of their participation in the selected group. Next, they were presented with three items pertaining to misinformation exposure, followed by corresponding questions regarding their agreement with the presented misinformation. Finally, participants completed the depression literacy measure.

The Ethics Committee of the university approved the study design. In total, we collected 493 responses with all variables of the present study filled out. 74 responses were eliminated from the dataset as participants signed “I do not remember” in the OCMHs they were participating in. Thus, they did not specify the OCMH, making it impossible to code an important variable for the study. In the case of double completion of the wave (n = 16 cases), the earliest wave was included in the study. The Ethics Committee of Università della Svizzera italiana approved the study design (CE_2021_10). All methods were performed in accordance with the Declaration of Helsinki. Informed consent was obtained from all participants.

### Measures

The survey’s items are reported in Additional file 1.

#### Misinformation exposure

The Italian scale was created ad hoc based on prior studies measuring susceptibility to misinformation. Participants were asked to rate how often they encountered three misinforming claims in the preceding 30 days. The three items were based on the results of a previous content analysis on the groups [31]. The response options were from 1 = “Never” to 5 = “Very often”. Other studies measured exposure similarly by asking participants how frequently they had seen or heard misinforming statements in the past 30 days e.g [70, 71]. We averaged scores on the three misinformation items into an overall index as in [72].

#### Misinformation agreement

Misinformation agreement was measured by asking the participants their degree of agreement with the same items presented in the Misinformation exposure variable (Likert scale: 1 = completely disagree, 7 = completely agree). In line with other researchers, an index score was created by taking the average of all item ratings e.g [73].

#### Depression literacy questionnaire

The Depression Literacy Questionnaire [52] assesses mental health literacy specific to depression. The questionnaire consists of 22 items which are true or false. Respondents can answer each item with one of three options – true, false, or don’t know. Each correct response received one point, and a sum score represented the extent of depression literacy (α = 0.74). The Italian version used in the study was validated (unpublished). Translation and back translation were conducted to confirm the scale’s accuracy and appropriateness. A bilingual expert panel composed of an expert in the topic and the two translators was convened to identify and resolve the inadequate expressions/concepts of the translation, as well as any discrepancies between the forward translation and the existing previous versions of the questionnaire. The questionnaire was pilot tested with a small sample of participants (n = 10) to ensure the clarity of the items. We then administered the questionnaire to a larger sample of participants (n = 286) and conducted a series of analyses to assess its reliability and validity. Internal consistency was assessed using Cronbach’s alpha, and we found good internal consistency for the scale (α = 0.74). We also examined the convergent validity of the scale by comparing it to well-established health literacy measured: the Newest Vital Sign (r = .135, p = .022) and the HLS-EU-Q16 (r = .231, p < .001).

#### Type of OCMHs participation

Participants marked from a list the number of OCMHs they participated in the last month preceding the survey. The first author checked the presence of expert moderators in the OCMHs mentioned during the survey. This led to creating a dummy coded variable in which 0 = peer-led only OCMHs participation, 1 = mixed participation, and 2 = expert-led only participation. Although mixed participation was not highly informative on the type of content moderation participants were exposed to, we decided to retain this level in the variable due to its popularity in the sample (15,9% see section Preliminary results) and excluding this category would have compromised the external validity of the study and limited our ability to capture the diverse experiences of individuals engaged in multiple types of online mental health communities.

#### Covariates

Covariates included were frequency of participation, gender and age. The frequency of participation in the groups was assessed using a 4-point scale, ranging from “rarely” (less than once a month) to “very often” (almost every day). The frequency of participation was based on previous studies used to measure the frequency of participation [42]. We controlled for the frequency of participation as misinformation agreement might be impacted by the amount of time they spend on OCMHs platforms [74].

Participants were also asked to indicate the gender they identified to (male, female, other, or prefer not to answer). Due to the scarcity of values in the other categories, the latest was the further dummy coded in 0 = other genders, 1 = male. Furthermore, previous research has shown that identifying with the male gender is associated with greater susceptibility to health misinformation [75] and men have higher reticence to seek professional mental health care services due to mental health stigma [76, 77]. As only two participants selected “other”, and one participant preferred not to respond, we grouped them together with the female responses, to distinguish them from the male category, which we expected to be more distinct. This approach aligns with previous studies [78, 79]. Furthermore, we included age as previous studies showed that older age was associated with less susceptibility to health misinformation [22].

### Data analyses

Descriptive statistics were calculated for all measures to assess the normal distribution and detect outliers. Scale reliability was assessed with Cronbach’s alpha for Depression Literacy. However, as its values are quite sensitive to the number of items in the scale, and having two short scales with three items (misinformation exposure and misinformation agreement), according to [80], we report the inter-item correlation range for the scales [81]. recommends an optimal range for the inter-item correlation of.15 to 0.50. A collinearity analysis was carried out to check the prerequisites for a regression analysis, which showed no signs of collinearity (tolerance factor > 0.10 and variance inflation factor < 10) [82]. No multivariate outliers were found according to Mahalanobis distance. SPSS 28.0 was used in our study for statistical analyses. The significance level was set at 0.05. We used PROCESS Model 3 [83] with 95% bias-corrected bootstrap confidence intervals (CIs, with 10,000 bootstrap samples) to test the model of moderated moderation. The effects of gender, age, and frequency were controlled by including them as covariates in the moderation analysis in the PROCESS macro. Following suggestions outlined in Hayes [68], mean centering was conducted on variables that defined products.

Continuous variables were standardized before conducting the model. As the variable Type of OCMHs participation had three levels we used the multi-categorical moderator function in PROCESS. To represent a multi-categorical moderator with k groups, PROCESS creates k − 1 variables and adds them to the model, in addition to k − 1 products representing the interaction [84]. Thus, two dummy variables were created to represent the Type of OCMHs participation and two interaction terms to represent the interaction between the type of OCMHs participation, depression literacy, and misinformation exposure.

## Results

### Preliminary results

Survey data were obtained from 403 participants. The sample consisted of 333 (82.6%) females, 67 males (16.6%), and 3 participants (0.7%) who responded with others. Participants were members of either a peer-led (n = 126, 31.3%), expert-led (n = 213, 52.9%), or both peer-led and expert-led OCMHs (‘mixed’ category, n = 64; 15,9%). The descriptive statistics, reliability coefficients, and Pearson correlations for the study variables are presented in Table 1.

**Table 1:** Descriptive statistics and correlations among variables in the whole sample (N = 403)

Variables	M (SD)	Skewness	Kurtosis	α/IIC	Age	Gender	Frequency	Misinformation Exposure	Misinformation Agreement	
Age (18–76)	42.35 (11.99)	0.13	− 0.73	NA	-					
Gender	0.17 (0.37)	NA	NA	NA	0.145**	-				
Frequency	3.16 (0.96)	− 0.83	− 0.446	NA	0.026	0.029	-			
Misinformation Exposure	2.46 (0.78)	0.093	− 0.173	0.18 − 0.42	0.068	− 0.017	0.128*	-		
Misinformation Agreement	2.32 (1.17)	1.03	0.71	0.20-0.33	0.001	0.109*	− 0.038	0.157**	-	
Depression Literacy	14.28 (3.58)	− 0.49	0.26	0.74	0.010	− 0.033	0.105*	0.118*	− 0.158**	

Note. *p < .05; **p < .01. Gender was dummy coded (male = 1; non-male = 0).

Participants were members of 64 groups, of which only five were moderated by health professionals (three psychotherapists, one psychologist, one nurse).

### Main results

We assessed a moderated-moderation model in which the moderation by depression literacy of the misinformation exposure effect is moderated by the type of OCMHs participation (see Table 2).

**Table 2:** Coefficients and 95% confidence intervals of moderated-moderation for predicting Misinformation Agreement

			β	SE	t	P
Constant			0.1529	0.1005	1.6892	0.0920
	**Focal antecedent**: Misinformation Exposure		0.3221***	− 0.0729	3.8808	0.0001
	Depression Literacy		− 0.2701**	0.0818	-3.2933	0.0011
	Depression Literacy x Misinformation Exposure		− 0.2179**	0.0508	-3.2217	0.0014
	Type of OCMHs participation (D1)		− 0.0174	0.1584	− 0.1529	0.8786
	Type of OCMHs participation (D2)		− 0.2322*	0.1179	-2.0741	0.0387
	Type of OCMHs participation (D1) x Misinformation Exposure		− 0.1031	0.1667	− 0.6224	0.5340
	Type of OCMHs participation (D2) x Misinformation Exposure		− 0.2451*	0.1063	-2.2436	0.0254
	Type of OCMHs participation (D1) x Depression Literacy x Misinformation Exposure		0.2711	0.1526	1.9528	0.0516
	Type of OCMHs participation (D2) x Depression Literacy x Misinformation Exposure		0.2497*	0.0861	2.4578	0.0144
**Covariates**						
	Gender		0.0896	0.0483	1.8802	0.0608
	Age		− 0.0292	0.0514	− 0.6278	0.5305
	Frequency of participation in OCMHs		− 0.0571	0.0509	-1.1650	0.2447
	**Test of conditional “Depression Literacy × Type of OCMHs participation” interaction at values of Misinformation Exposure**					
	**Depression literacy**	**Type of OCMHs participation**	**β**	**SE**	**LLCI**	**ULCI**
	-1 SD	Peer-led	0.5338***	0.1024	0.3324	0.7351
	-1 SD	Mixed-led	0.1699	0.1670	− 0.1584	0.4983
	-1 SD	Expert-led	0.0411	0.1050	− 0.1653	0.2474
	Mean Depression Literacy	Peer-led	0.3182***	0.0820	0.1570	0.4794
	Mean Depression Literacy	Mixed-led	0.2254	0.1243	− 0.0189	0.4697
	Mean Depression Literacy	Expert-led	0.0758	0.0705	− 0.0627	0.2144
	+ 1 SD	Peer-led	0.1026	0.1092	− 0.1121	0.3172
	+ 1 SD	Mixed-led	0.2809	0.1814	− 0.0758	0.6376
	+ 1 SD	Expert-led	0.1030	0.1030	− 0.0919	0.3131

Note. *p < .05 **p < .01 ***p < .001. Gender was dummy coded (male = 1; non-male = 0).

D1 coding: peer-led OCMHs = 0, mixed OCMHs = 1, expert-led OCMHs = 0.

D2 coding: peer-led OCMHs = 0, mixed OCMHs = 0, expert-led OCMHs = 1.

### Hypothesis 1

Misinformation exposure predicted a larger Misinformation agreement (β = 0.3221, t = 3.8808, p < .001).

### Hypothesis 2

Depression literacy predicted lower misinformation agreement (β = -0.2701, t =-3.2933, p = .0011). The positive association between misinformation exposure and misinformation agreement was moderated by depression literacy (β = -0.2179, t = -3.2217, p = .0014).

### Hypothesis 3

Participating in expert-led OCMHs predicted lower misinformation agreement (contrast: peer-expert; β = -0.2351, t = -2.0741, p = .0387). The positive association between misinformation exposure and misinformation agreement was moderated by type of OCMHs participation as the contrast interaction peer-expert OCMHs had a significant effect (β = − 0.2322, t =-2.2436, p = .0254).

### Research question

**Fig. 2:**
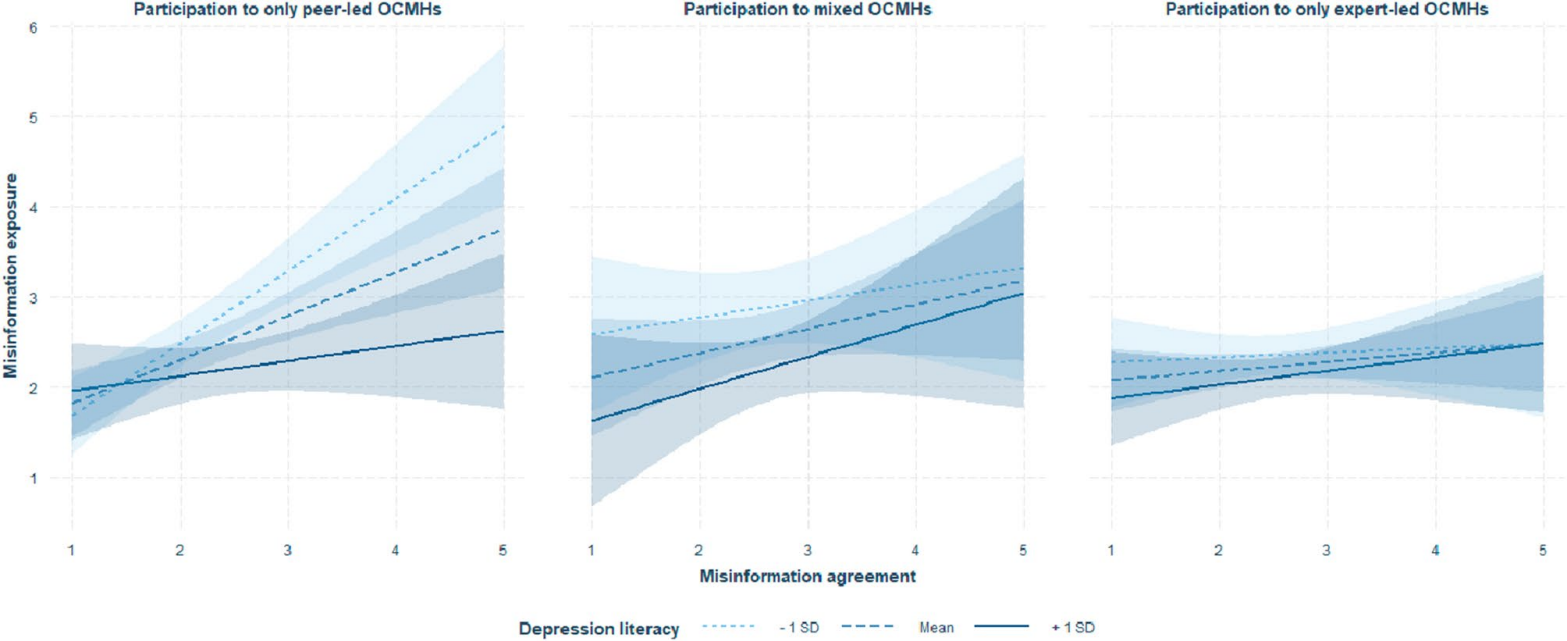
Plot representing the three-way interaction

**Fig. 3:**
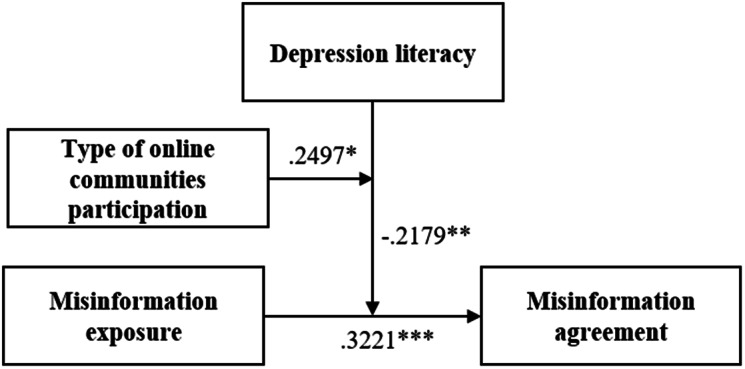
Model with coefficients (contrast: peer-expert; *p < .05 **p < .01)

The three-way interaction between misinformation exposure, depression literacy, and type of OCMHs participation in misinformation agreement was also significant (ΔR^2^ = 0.02; F(2,387) = 3.8037, p = .0231). The bootstrap CIs indicated significant effects (p < .001) for 2(of 3) levels of depression literacy: low (β = 0.5338) and medium (β = 0.3182), with an effect significant only in peer-led communities. See Figs. 2 and 3.

## Discussion

The present study provides compelling evidence that individuals, when repeatedly exposed to misinformation, are more likely to agree with it (H1), which aligns with the theoretical framework of the illusory truth effect [69]. This first result is concerning as members look for information on online communities to make important health decisions [85]. However, the study also found that individuals with higher levels of depression literacy were better equipped to resist mental health-related misinformation (H2), consistent with prior research [46–48]. Therefore, we can conclude that depression literacy is an individual difference that impacts the probability of agreeing (or not) with the misinformation people are exposed to, highlighting the importance of health literacy education to mitigate the effects of misinformation exposure.

The second buffer: the type of OCMHs participation was not only a moderator of the association Misinformation exposure-misinformation agreement (H3), with participants in expert-led OCMHs having lower chances of agreeing to misinformation, but also a moderator of the moderation between depression literacy and misinformation exposure on misinformation agreement (RQ). Specifically, members of peer-led OCMH groups with higher levels of depression literacy were almost as susceptible to misinformation as those in expert-led OCMH groups. Conversely, those with lower levels of depression literacy and lacking the external help of experts in peer-led OCMH groups had the worst outcomes. Future studies should primarily address these vulnerable members of OCMHs, which we identified by combining the analysis of internal (literacy) and external resources (type of participation).

The findings highlight the importance of expert sources to correct health misinformation in the social media [86]. Healthcare professionals, in particular, play a critical role in countering misinformation, as suggested by Schulz & Nakamoto [87], and in line with previous studies, are integral to the success of online communities as they are equipped with the required professional health knowledge to offer trustworthy advice on the causes, prevention, and treatment of disease [88].

One possible strategy to diminish misinformation exposure is to encourage health professionals to administer OCMHs to ensure accurate and trustworthy information and explore possible incentives to promote their involvement or tools that might contribute to their role in the communities, such as automated moderation. Another avenue for future research is to examine the factors that influence individuals to participate in either peer-led or expert-led communities and the perceived differences between these types of communities. Furthermore, gaining insight into the mechanisms underlying the potential positive outcomes associated with participation in expert-led OCMHs can contribute to enhancing the quality of peer-led communities and fostering improved regulation within these virtual spaces.

Furthermore, as suggested by [64], through their participation in OCMHs, health professionals might also inform their professional practice about the misinformation and ambiguous discourses regarding symptoms or treatments. To conclude, while they represented a minority compared to other participants, future studies should better explore members’ participation patterns in both peer-led and expert-led communities (‘mixed’ category). This will provide a more thorough understanding of their experiences and the potential risks they may face.

### Limitations and future directions

While our study offers valuable insights into the relationship between exposure to misinformation and mental health misbeliefs, several limitations need to be considered. Firstly, given the cross-sectional nature of the employed data, causative associations between exposure to misinformation and mental health misbeliefs could only be speculated here. Future longitudinal studies could clarify the temporal ordering of variables investigated by examining the cross-lagged paths. Specifically, such studies can help determine whether participants’ beliefs (i.e. agreement with misinformation) precede their volume of exposure to health misinformation or the OCMHs (peer- or expert-led) they decide to participate to and shed light on how the dynamics of misinformation exposure and agreement unfold over time.

Secondly, our study used a self-selection sampling method, limiting our results’ generalizability to larger populations. Future studies should use probability sampling methods to address this limitation.

Thirdly, with respect to limitations regarding the measurement of the variables investigated, our study only measured the volume of misinformation exposure and did not account for the amount of misinformation correction. This is an important variable that should be included in future studies to fully understand the impact of misinformation exposure on mental health misbeliefs.

Furthermore, our use of a depression literacy scale only measured declarative knowledge and may not have captured the full complexity of the literacy construct. Future studies should complement this by investigating the role of critical skills. Another limitation is the potential for recall bias as our measures of misinformation exposure were based on self-reported past experiences. While the specific nature of the questions minimized this bias, future studies should consider using more objective measures of exposure to misinformation. Previous studies have shown that confirmation bias might influence memory and recall, such as that participants in our study could have had a better memory for instances consistent with their prior beliefs [89].

About the frequency measure included as a covariate, it is important to acknowledge that it provides a general indication of participation but lacks specificity, particularly in mixed groups where it does not distinguish whether members attended more peer- or expert-led OCMHs. To obtain a more accurate measurement, future studies should consider inquiring about the frequency of participation for each OCMH or request participants to rank the OCMHs in terms of their level of involvement.

We also acknowledge that, as mental health misinformation exposure and agreement were measured with ad hoc scales, although the items were created from a content analysis, some items were too vague or exhibited a double-barreled issue as there might be differences in exposure/agreement to the different kinds of pharmacological and psychological therapies. However, with the exclusion of item 1, as suggested by reviewers, results were similar to the ones found with all the 3 items. Future studies should address separately the different kinds of treatments. In general, there is a need to improve the operationalization of these variables considering that online people might be exposed to varying types of mental health misinformation.

Lastly, we recognize that there might be heterogeneity in the moderation of OCMHs, even among experts. Future studies should explore the quality (debunking, scientific dissemination, censuring) and quantity of moderation in OCMHs as a continuum rather than a dichotomy.

## Conclusion

The study examined responses from 403 members of Facebook OCMHs and found that the vaster the quantity of misinformation regarding mental health the people were exposed to, the more they were likely to believe in it. Therefore, efforts are necessary to reduce exposure to health misinformation and interventions to reduce its impact. Depression literacy was found to have a mitigating effect, suggesting that improving depression literacy could serve as a public health goal to counter the negative impact of misinformation on mental health outcomes. Moreover, the study highlighted the potential role of the type of participation in OCMHs (expert- vs. peer-led) in reducing misbeliefs. It emphasized the importance of health professionals in OCMHs and a resource to incentivize. Findings have significant implications for understanding the complex interplay between the variables investigated and informing interventions and policies to reduce misinformation’s negative impact on mental health, especially for the most vulnerable members identified.

### Electronic supplementary material

Below is the link to the electronic supplementary material.

Supplementary Material 1

## Data Availability

The datasets used and analysed during the current study available from the corresponding author on reasonable request.

## List of abbreviations

OCMH: Online community for mental health
