# Supplementary material for: Buffering against exposure to mental health misinformation in online communities on Facebook: the interplay of depression literacy and expert moderation
Source: BMC Public Health. 2023 Aug 18;23:1577. doi: 10.1186/s12889-023-16404-1 (PMC10436646; doi:10.1186/s12889-023-16404-1)
Supplement: Supplementary file 1 — Supplementary Material 1 [file 12889_2023_16404_MOESM1_ESM.docx]

**Additional File 1: Measures**

**Misinformation Exposure**

Items:

1. Medication or psychotherapy does not cure mental disorders.
2. Psychologists/psychotherapists only care about making money from patients.
3. It is preferable to use natural therapies (supplements, homeopathy, naturopathy) than to take psychotropic drugs.

Response options: 1 = Never, 2 = Rarely, 3 = Sometimes, 4 = Often, 5 = Very often.

**Misinformation Agreement**

Items:

1. Medication or psychotherapy does not cure mental disorders
2. Psychologists/psychotherapists only care about making money from patients.
3. It is preferable to use natural therapies (supplements, homeopathy, naturopathy) than to take psychotropic drugs.

Response options: 1 = Completely disagree; 7 = Completely agree

**Depression literacy questionnaire (22 items)**

Response options: True, False, I do not know

| **Original version** | **Italian translation**  **(Scala di Alfabetizzazione sulla Depressione)** |
| --- | --- |
| 1. People with depression often speak in a rambling and disjointed way. (False) | Le persone che soffrono di depressione parlano spesso in modo sconclusionato e sconnesso. |
| 1. People with depression may feel guilty when they are not at fault. (True) | Le persone che soffrono di depressione possono sentirsi colpevoli anche quando non hanno colpe. |
| 1. Reckless and foolhardy behaviour is a common sign of depression. (False) | Un comportamento spericolato e imprudente è un segno comune di depressione. |
| 1. Loss of confidence and poor self-esteem may be a symptom of depression. (True) | La perdita di fiducia e la scarsa autostima possono essere sintomi di depressione. |
| 1. Not stepping on cracks in the footpath may be a sign of depression. (False) | Non calpestare le righe sul pavimento può essere un segno di depressione. |
| 1. People with depression often hear voices that are not there. (False) | Le persone con depressione spesso sentono voci che non ci sono. |
| 1. Sleeping too much or too little may be a sign of depression. (True) | Dormire troppo o troppo poco può essere un segno di depressione. |
| 1. Eating too much or losing interest in food may be a sign of depression. (True) | Mangiare troppo o perdere interesse per il cibo può essere un segno di depressione. |
| 1. Depression does not affect your memory and concentration. (False) | La depressione non influisce sulla memoria e sulla concentrazione. |
| 1. Having several distinct personalities may be a sign of depression. (False) | Avere personalità multiple può essere un segno di depressione. |
| 1. People may move more slowly or become agitated as a result of their depression. (True) | Le persone possono muoversi più lentamente o diventare agitate come risultato della loro depressione. |
| 1. Clinical psychologists can prescribe antidepressants. (False) | Gli psicologi clinici possono prescrivere antidepressivi. |
| 1. Moderate depression disrupts a person’s life as much as multiple sclerosis or deafness. (True) | La depressione moderata disturba la vita di una persona tanto quanto la sclerosi multipla o la sordità. |
| 1. Most people with depression need to be hospitalised. (False) | La maggior parte delle persone con depressione deve essere ricoverata in ospedale. |
| 1. Many famous people have suffered from depression. (True) | Molti personaggi famosi hanno sofferto di depressione. |
| 1. Many treatments for depression are more effective than antidepressants. (False) | Molti trattamenti per la depressione sono più efficaci degli antidepressivi. |
| 1. Counselling is as effective as cognitive behavioural therapy for depression. (False) | La consulenza psicologica è efficace tanto quanto la terapia cognitivo comportamentale per la depressione. |
| 1. Cognitive behavioural therapy is as effective as antidepressants for mild to moderate depression. (True) | La terapia cognitivo comportamentale è efficace quanto gli antidepressivi per la depressione lieve o moderata. |
| 1. Of all the alternative and lifestyle treatments for depression, vitamins are likely to be the most helpful. (False) | Di tutti i trattamenti alternativi e di stile di vita per la depressione, le vitamine sono probabilmente le più utili. |
| 1. People with depression should stop taking antidepressants as soon as they feel better. (False) | Le persone depresse dovrebbero interrompere l'assunzione di antidepressivi non appena si sentono meglio. |
| 1. Antidepressants are addictive. (False) | Gli antidepressivi creano dipendenza. |
| 1. Antidepressant medications usually work straight away. (False) | I farmaci antidepressivi di solito funzionano immediatamente. |

**Frequency of participation**

Response options:

1. Rarely (I visited the groups less than once a month)

2. Sometimes (I visited groups at least once a month)

3. Often (I visited the groups at least once a week)

4. Very often (I visited the groups almost every day)
